# Economic Evaluation of Pharmacist-Led Digital Health Interventions: A Systematic Review

**DOI:** 10.3390/ijerph191911996

**Published:** 2022-09-22

**Authors:** Taehwan Park, Hyemin Kim, Seunghyun Song, Scott K. Griggs

**Affiliations:** 1 Pharmacy Administration and Public Health, College of Pharmacy and Health Sciences, St. John’s University, Queens, NY 11439, USA; 2 College of Pharmacy and Health Sciences, St. John’s University, Queens, NY 11439, USA; 3 Pharmacy Administration, University of Health Sciences and Pharmacy in St. Louis, St. Louis, MO 63110, USA

**Keywords:** economic evaluation, cost-effectiveness, pharmacists, digital interventions, technology

## Abstract

There has been growing interest in integrating digital technologies in healthcare. The purpose of this study was to systematically review the economic value of pharmacist-led digital interventions. PubMed, Web of Science, and the Cochrane databases were searched to select studies that had conducted economic evaluations of digital interventions by pharmacists for the period from January 2001 to February 2022. Economic evidence from 14 selected studies was synthesized in our analysis. Pharmacists used telephones, computers, web-based interventions, videotapes, smartphones, and multiple technologies for their digital interventions. Prior studies have reported the results of telephone-based interventions to be cost-effective. Alternatively, these interventions were found to be cost-effective when reevaluated with recently cited willingness-to-pay thresholds. In addition, pharmacist-led interventions based on computers, web-based interventions, smartphones, and multiple technologies have been reported to be cost-effective in previous studies. However, videotape-based intervention was found cost-ineffective because there was no significant difference in outcomes between the intervention and the usual care groups. If this intervention had been intensive enough to improve outcomes in the intervention group, favorable cost-effectiveness results could have been obtained. The economic evidence in the previous studies represented short-term economic values. Economic evaluations of the long-term value of digital interventions are warranted in future studies.

## 1. Introduction

Digital health is a discipline that applies software and hardware to enhance health and health care delivery [1,2]. According to the FDA, the scope of digital health widely includes mobile health, telehealth (or telemedicine), wearable devices, and health information technology [3]. Recent advances in digital technology offer the opportunity to broaden the reach of healthcare. For example, increasing numbers of individuals in rural areas with limited access to healthcare can now receive timely interventions from remotely located healthcare providers. Multiple physicians can easily access patient charts, communicate with patients, and monitor disease management using their smartphone, mobile applications, and accessible software [4,5,6,7,8]. Pharmacists also use digital technology in their practices. They used telephones, mobile applications, and a remote monitoring device to improve medication adherence and drug-related outcomes, to educate patients, and to monitor patient symptoms remotely [9,10,11]. Pharmacists also provided computer- or web-based interventions to improve hypertension management [12] and to reduce medication errors and adverse events [13,14]. Sometimes, they used multiple technologies in their interventions [15,16]. Specifically, pharmacists offered face-to-face consultation (based on either a web-based program or a technology platform) plus telephone follow-up to communicate with patients, to educate patients, and to manage their health-related conditions in an effective manner. Because of the benefits conferred by technology, digital interventions have gained increasing popularity in healthcare.

By providing information of the incremental impact on economic and health outcomes as compared with alternative interventions (e.g., face-to-face usual care), economic evaluations of digital health interventions can guide practitioners or policy makers on whether to adopt such interventions. Digital interventions may be expected to bring economic benefits, for example, by reducing transportation costs, by decreasing time to diagnosis, and by making it possible for patients to remain in their homes rather than resorting to costly health care facilities [17,18]. In contrast, these interventions may prove costly because of the high upfront expenses for implementation, ongoing costs for support and maintenance, increased expense for added personnel interventions, and their resource-intensive nature [19,20]. Therefore, uncertainty exists about whether technology-based interventions are cost-effective. For this reason, studies have examined the economic value of digital interventions such as telehealth/telemedicine, electronic health (e-health), and mobile health (m-health) [21,22,23,24,25,26,27]. These studies have shown mixed results on the cost-effectiveness of digital interventions. Economic value of the interventions has varied, for example, by study populations, types of interventions, comparison groups, and outcome measures. Of note, all of these studies have focused primarily on physicians’ interventions for specific population groups such as patients with diabetes [23,24], somatic conditions [25], or mental illness [26], and adolescents with chronic conditions [27]. To date, no studies have reviewed the cost-effectiveness of pharmacist-led digital interventions in general, regardless of disease type. Therefore, questions remain as to the potential economic impact that pharmacists would be able to exert on patients’ outcomes. Accordingly, the objective of this systematic review was to synthesize the economic evidence of digital interventions provided by pharmacists for their patients. Our review provides economic evidence for multiple digital technologies that can help practitioners make decisions in adopting these technologies. In addition, such evidence can contribute to a better understanding of the benefits of extensive investment of these digital tools. Information from economic evaluation can be also used to make coverage and reimbursement decisions. 

## 2. Materials and Methods

### 2.1. Search Strategy

A systematic literature search was conducted to identify all published articles assessing the cost-effectiveness of pharmacist-led digital interventions using databases such as PubMed, Web of Science, and the Cochrane database of Systematic Reviews for the period from January 2001 to February 2022. Here, digital health was operationally defined as the use of information and communication technologies to improve health, and thus, it included categories such as mobile health, telehealth, electronic health as well as wearable devices and any other tools that could be used to enhance health. To guide the search strategy, the following patient, intervention, comparison, outcome (PICO) framework was developed:

P: Adult patients
I: Digital intervention(s) by pharmacists
C: Usual care (i.e., comparator treatment in published studies)
O: Health-related clinical outcomes from patients

The Medical Subject Heading (MeSH) terms and keywords for pharmacist-led digital interventions (pharmacist digital intervention, pharmacist mobile health intervention, pharmacist telehealth, pharmacist telecare, pharmacist teleconsult*, pharmacist telecommunication, telepharmacy, pharmacist electronic health, pharmacist ecommunication, pharmacist remote consultation, pharmacist short message service, pharmacist text messag*, pharmacist internet consultation, pharmacist internet monitoring, pharmacist communicate*, pharmacist video consultation, pharmacist vide monitoring, pharmacist video communicate*, pharmacist mobile application intervention) were coupled with those terms meaning economic evaluation (comparative effectiveness, economic evaluation, economic modeling, cost-benefit, cost-effectiveness, cost utility, cost-saving*, pharmacoeconomics). The search was limited to full-text articles published in English. 

### 2.2. Inclusion/Exclusion Criteria and Selection Process

**Figure 1:**
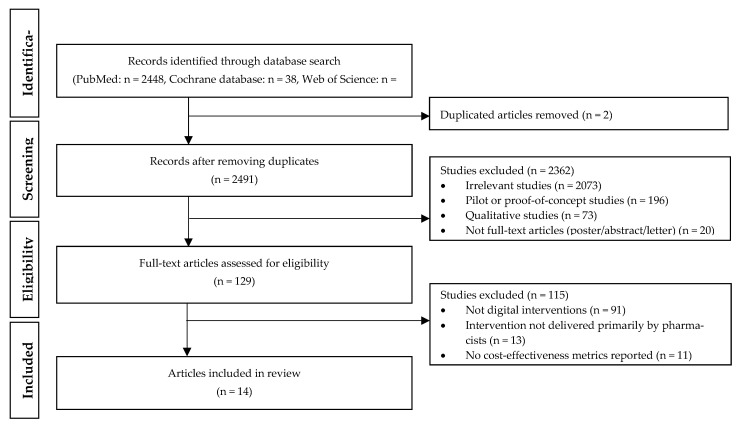
Flow diagram of the search strategy and selection of articles.

Figure 1 depicts a flow diagram showing the selection process. The search strategy identified 2448, 38, and 7 articles from PubMed, the Cochrane database, and Web of Science, respectively. Removing duplicates left a total of 2491 unique articles. The titles and abstracts of these articles were reviewed in the first level of screening. Articles were excluded if they were (a) irrelevant to the purpose of this review; (b) pilot or proof-of-concept studies; (c) qualitative studies; and (d) not full text articles (e.g., poster, abstract, or letter). This screening process resulted in the exclusion of 2362 articles. The remaining 129 articles were then assessed for eligibility. Articles were excluded if (a) no digital interventions were provided to patients; (b) interventions were not delivered primarily by pharmacists; and (c) no cost-effectiveness metrics (e.g., incremental cost-effectiveness ratio) were reported. After applying these exclusion criteria, a total of 14 articles remained and included in our analysis.

### 2.3. Quality Assessment of the Studies

The Quality of Health Economics Studies (QHES) instrument, a validated tool to assess the quality of economic studies [28], was used to evaluate the quality of the 14 selected studies. As presented in Table 1, this instrument includes 16 items, each of which has a weighted score ranging from 1 to 9. The sum of the total item scores ranges from 0 (lowest quality) to 100 (highest quality). Using the QHES instrument, the quality of the studies included in this review was determined as poor (QHES score < 50), fair (50 ≤ QHES score < 75), or good (75 ≤ QHES score ≤ 100), as suggested by prior studies [29,30,31]. 

Two reviewers (H-M.K. and S.S.) independently extracted data from the 14 studies and evaluated their quality. The two reviewers showed a high level of agreement (ranging from 82.1% to 95.5%) in extracting data and evaluating the quality of the studies. Any discrepancies were discussed with a third reviewer (T.P.) to reach consensus. 

## 3. Results

### 3.1. Overview of the Included Studies

Table 2 shows an overview of the characteristics of the 14 studies included in our review. Of the 14 studies, cost-effectiveness analysis (n = 6) and cost–utility analysis (n = 6) were the most frequently used types of economic analyses. These studies were conducted in seven different countries including the U.S. (n = 5), Spain (n = 3), and Australia (n = 2). Various perspectives (patient, payer, and societal) were adopted in the studies. The majority of the studies (n = 9) used data directly obtained from clinical trials rather than a model-based approach for their economic analysis. The time horizon employed in the economic analysis spanned from 14 days to lifetime. Most studies (n = 12) received funding from national level granting agencies and industries, whereas two studies [32,33] received no funding.

### 3.2. Telephone-Based Intervention

Table 3 presents the summary of the 14 included studies. Of these 14 studies, pharmacists used a telephone-based intervention in seven. Five out of the seven studies reported the economic benefits of the phone interventions [32,33,35,36,38]. Dehmar et al.’s CEA study revealed that home blood pressure monitoring and pharmacist case management improved hypertension care in a cost-effective manner [35]. They reported that this intervention cost USD 7337 per person achieving hypertension control. Another CEA study by Faleh AlMutairi et al. found telemedicine-based care to be cost-effective for patients with type 2 diabetes [32]. They reported an incremental cost-effectiveness ratio (ICER) of SAR 2373 per 1% reduction in HbA1c. In a CBA study with polymedicated elderly patients admitted to an internal medicine ward, a clinical pharmacist carried out phone-based follow-up interventions after patients’ discharge [36]. The interventions achieved a net cost saving of EUR 1301 by reducing readmissions of these patients. The net cost savings became greater in those with intermediate and high risks of potentially avoidable readmission. A CMA study was conducted using a pre- and post- design in which patients with HIV received pharmacist teleconsultations [33]. The economic evaluation of the teleconsultation interventions showed EUR 137 patient/year costs-saved and 18.5 h/patient/year working time gained. In Padwal et al.’s CUA study, patients with cerebrovascular condition were provided home blood pressure telemonitoring services with pharmacist case management interventions [38]. Their results using a Markov model demonstrated that telemonitoring with case management was dominant (i.e., less costly and more effective) compared with usual care. In contrast to these five studies, telemedicine-based care was not consistently considered to be cost-effective in two studies [19,20]. In their CUA study, Painter et al. included rural veterans with posttraumatic stress disorder (PTSD) assigned to either telemedicine care or usual care [19]. Although the telemedicine care intervention was more effective than usual care with an incremental increase in QALY of 0.008, the intervention was relatively expensive with an incremental cost of USD 2495, resulting in an ICER of USD 185,565/QALY. Therefore, the improvement in QALY did not translate to economic benefit due to the high cost of this intervention given the conventionally reported willingness-to-pay (WTP) thresholds of USD 50,000–USD 150,000/QALY. Of note, their subgroup analyses focusing on patient subgroups with anxiety disorder, depression, and panic disorder showed that the intervention was dominant (i.e., produced greater QALY at lower costs) (data not shown in Table 3). In Pyne et al.’s CUA study, patients with depression were randomly assigned to receive either telemedicine care or usual care [20]. In their base-case analysis including outpatient costs alone, the incremental intervention effects on expenditures (β = USD 1528, *p* < 0.001) and QALY (β = 0.018, *p* = 0.04) were significant, resulting in an ICER of USD 85,634/QALY. The ICERs ranged from USD 111,999 to USD 132,175/QALY in their secondary analysis, where inpatients costs were also included in addition to outpatient costs. The ICERs exceeded a WTP threshold of USD 50,000/QALY, indicating that telemedicine care might not be cost-effective given this threshold. 

### 3.3. Computer- or Web-Based Intervention

Three CEA studies performed an economic evaluation of computer- or web-based interventions by pharmacists [12,13,14]. All these studies found the interventions to be cost-effective. In Avery et al.’s trial, patients given electronically prescribed prescriptions by a general practitioner were assigned to either the control group who received computer-generated simple feedback or the intervention group who received a pharmacist-led intervention composed of feedback, educational outreach, and dedicated support [13]. The results of the study showed that the ICER of the intervention was GBP 66 per error avoided, indicating the intervention was cost-effective. In another trial by Fishman et al., patients with hypertension were randomized to receive either usual care plus a home blood pressure (BP) monitor or a pharmacist’s web-based management intervention plus a home BP monitor [12]. The intervention was found to be cost-effective with ICERs of USD 2220 and USD 1850 per life year for women and men, respectively. In Hope et al.’s study, a tiered review method (computer-based review prior to a clinician’s review) was compared with the traditional pharmacist-based review approach, both of which were used to identify adverse drug events (ADEs) and medication errors (MEs) in adults with outpatient care at ambulatory clinics [14]. This tiered review turned out to be more cost-effective than the traditional review (USD 42 vs. USD 69 per ADE identified, respectively).

### 3.4. Videotape-Based Intervention

Bosmans et al. conducted a randomized clinical trial in which subjects comprised those who had visited a pharmacy with a new prescription for a non-tricyclic antidepressant [34]. The subjects were randomly assigned to either the coaching program (consisting of three contacts with the pharmacist and a take-home video) or usual care. Outcome measures were treatment adherence and improvements in depressive symptoms measured using the Hopkins Symptom Checklist (SCL). Although the coaching program cost slightly more than usual care (EUR 3275 vs. EUR 2961), there were no significant differences in outcomes. Therefore, the authors concluded that the program was not cost-effective compared with usual care. 

### 3.5. Smartphone-Based Intervention

Lowres et al. evaluated the economic value of pharmacy-based screening for unknown atrial fibrillation (AF) using an iPhone electrocardiogram (iECG) [37]. Compared with no screening strategy, screening with iECG was found to be cost-effective, with the ICERs of AUD 5988 per QALY and AUD 30,481 for preventing one stroke. 

### 3.6. Multiple Techologies-Based Intervention

Two CEA studies conducted economic evaluations of multiple technologies-based interventions [15,16]. A clinical trial by Amador-Fernandez et al. included patients who presented with minor ailments or requested a non-prescription drug for such ailments [15]. The patients were randomized to receive either the intervention (comprising face-to-face consultation utilizing a web-based program plus telephone follow-up) or usual care. The intervention was considered to be cost-effective with an ICER of EUR 24,733/QALY given a WTP threshold of EUR 25,000/QALY. Those with minor ailments were also recruited in Dineen-Griffin et al.’s study [16]. The intervention group received face-to-face consultation using the technology-integrated platform plus telephone follow-up, whereas the control group received usual care alone. The intervention was found to be cost-effective with an ICER of AUD 2277/QALY.

### 3.7. Study Quality 

Table 4 summarizes the results of quality evaluation using the QHES instrument (actual points in Table 4 are available upon request). Overall, the quality of the included studies was good (57%) or fair (43%). No study was rated as poor. 

## 4. Discussion

Despite growing interest among pharmacists in using digital technology in practice, there has been a paucity of research about the economic evaluations of digital interventions. Accordingly, the purpose of the current study was to systematically review the economic value of pharmacist-led digital interventions. Pharmacists used telephones with call functions only, computers, web-based interventions, videotapes, smartphones, or multiple technologies (such as web plus telephone or technology platform plus telephone) for their digital interventions. First, telephone-based interventions were generally found to be cost-effective in the studies included in this review. That is, five out of the seven phone-based studies revealed the economic benefits of the phone interventions [32,33,35,36,38]. In the other two telephone-based studies by Pyne et al. and Painter et al. [19,20], the authors concluded that the interventions were not cost-effective because the ICERs of the interventions were higher than the conventionally cited WTP thresholds of USD 50,000–USD 150,000/QALY. However, these thresholds were proposed more than several decades ago. If these studies were reevaluated using more recently suggested thresholds such as USD 200,000/QALY or USD 300,000/QALY [39,40], these interventions could be economically favorable. Specifically, all ICERs reported in Pyne et al.’s study fall below the thresholds of USD 200,000–USD 300,000/QALY, which makes the intervention cost-effective. Similarly, the ICER from base-case analysis in Painter et al.’s study is lower than the threshold of USD 200,000– USD 300,000/QALY. Additionally, their subgroup analyses showed cost-savings of the intervention. That is, this intervention resulted in greater QALYs at lower costs among those with anxiety disorder, depression, or panic disorder. As such, Painter et al.’s findings suggest that phone intervention could be economically sound for individuals with comorbid mental disorders. This implies the importance of targeting specific population groups. It is crucial that clinicians, researchers, and policy makers identify population groups who can benefit most from digital interventions and consider prioritizing their needs in providing such interventions. Next, a pharmacist’s computer- or web-based interventions were consistently found to be cost-effective in earlier studies [12,13,14]. However, in Bosmans et al.’s study, providing videotape-based intervention to patients with depression was found not cost-effective [34]. Because there were no significant differences in treatment adherence and depressive symptoms between the intervention and usual care groups, the intervention was considered an economically less preferred option. Of note, this study adopted a 6-month time horizon, and thus, the long-term effects of this intervention were not evaluated. Moreover, as the authors stated, their results were not consistent with those of two other studies reporting significant improvements in adherence to antidepressants after pharmacist interventions [41,42]. According to Bosmans et al., their intervention was not intensive—i.e., they deliberately delivered a minimal easy-to-implement intervention intended not to interrupt the pharmacist’s daily practice and to minimize participant attrition. This could possibly explain the non-significant difference in adherence between the intervention and usual care groups. In the two other studies that showed significantly improved adherence in the intervention group, pharmacist interventions were more intensive. Perhaps, making the interventions in Bosmans et al.’s study intensive enough to improve treatment adherence would have led to favorable cost-effectiveness results. Therefore, the cost-effectiveness results of the pharmacist’s interventions may be dependent upon how the interventions are designed. The degree of the pharmacist’s engagement is likely to affect patient outcomes, which in turn influences the economic value of the intervention. Even though pharmacists provide digital interventions, if they are engaged to a limited extent in using technologies, the interventions may not improve patient outcomes as effectively as expected. This highlights the importance of pharmacists’ dynamic use of digital tools to enhance patient outcomes. In addition, the smartphone-based intervention was considered cost-effective in a prior study [37]. Finally, two previous studies using multiple technologies revealed that interventions based on such technologies were cost-effective [15,16]. 

There may be several possible reasons for why technology-based interventions are cost-effective. First, patients who receive the intervention can save direct nonmedical costs (e.g., transportation or childcare costs) or indirect costs (e.g., productivity loss), for example, by receiving care in a remote location rather than in costly care facilities. Alternatively, early identification/detection of a disease using technology can prevent disease progression and costly health outcomes. For example, in one of the studies included in our review, the authors screened the elderly to identify new AF using iECG [37]. Those identified could have received prompt treatment to prevent stroke and to thus avoid the high cost burden of stroke. Such economic benefits can be obtained not only from preventing stroke but also from a wide range of conditions using a technology-based screening tool. Additionally, technology may enhance care providers’ efficiency. Using the technologies, clinicians can decrease their time for each patient. This will ultimately lead to reduction in costs to clinicians themselves as well as to society.

The current study found that telephoning was the most frequently used intervention tool among pharmacists. This finding is consistent with the results from other studies [9,10]. Nine out of the 14 studies included in this review (64.3%) adopted a telephone—either as a single digital intervention tool or combined with another digital tool. Similarly, a telephone was used most frequently as a digital intervention tool in 69.2% of the community pharmacists’ interventions [9] and 78.9% of the clinical pharmacists’ interventions [10]. Notably, computers or web-based interventions were used as intervention tools in only three studies. A smartphone was selected only in one study. Other tools such as a wearable device or social media were not adopted in the studies we reviewed. As noted in our earlier study [10], the telephone represents the traditional method of digital interventions. Pharmacists can consider more novel and diverse technologies in their interventions to serve expanded purposes. For example, smartphone technologies have been used for smoking cessation [43,44], weight management [45], and treatment of eating disorders [46]. In addition, wearable sensors integrated with smartphone apps record an individual’s real-time vital signs and health-related parameters, demonstrating the potential for personalized care solutions [47,48]. Pharmacists using these technologies to promote healthy behavior and better disease management, as noted in the examples, should result in positive outcomes in their patients. Once identifying how much these technologies can improve patient outcomes, the economic value of the interventions based on the technologies should be evaluated. 

There have been challenges in conducting economic analyses in prior studies. Although pharmacist-led digital interventions were found to be generally cost-effective in the studies, these results were limited to short-term economic values in most cases. A total of 11 studies (78.6%) chose a very short time frame (e.g., a time horizon ≤ 12 months), and the economic value was evaluated with this time frame. Therefore, the long-term economic value of digital interventions remains unclear. Furthermore, in most studies, the economic value was evaluated without using an economic model-based approach. One of the benefits of using an economic model is estimating the future costs and outcomes, thereby facilitating the evaluation of long-term economic value. These prior studies did not likely use an economic model because they adopted such a short time frame. Moreover, some studies did not include direct nonmedical and/or indirect costs in their analyses. However, technology can significantly save time and resources and thus reduce such direct nonmedical and indirect costs. In this sense, economic benefits of digital interventions could be estimated more accurately by including these costs with a societal perspective. In future studies to evaluate the economic value of digital interventions, researchers need to consider using an economic model (e.g., a Markov model) incorporating a sufficiently long time frame and perspective by including the comprehensive types of costs. 

Guidelines for evaluating internal and external validity in systematic reviews have been proposed by Avellar et al. [49]. According to the guidelines, authors of systematic reviews can report the important elements of internal and external validity such as information related to effectiveness, generalizability, applicability, and feasibility of an intervention. Thus, we abstracted such information from the studies and presented them as follows. First, this review included a wide range of population groups such as adults with minor ailments, diabetes, hypertension, HIV, cerebrovascular conditions, depression, elderly using ≥ 2 medications, and veterans with PTSD. Because a summary measure in the economic evaluations is an ICER, we were not able to calculate numerical impact estimates (e.g., regression coefficients, odds ratio, and adjusted mean differences), effect sizes, or p-values of the impact estimates. Additionally, interventions included in our review were pharmacist-led digital interventions using telephones, computers, webs, videotapes, smartphones, and multiple technologies. In general, pharmacists needed experience and education to implement such digital interventions. The comparison group in the studies usually received usual care or traditional care. All the information here should be considered in generalizing the study findings to a broader situation. 

This is the first systematic review of the cost-effectiveness of pharmacist-led digital interventions. Our review provides a narrative integrating the economic outcomes for multiple digital technologies that may inform decision-making for clinicians and policy makers. However, our study has several limitations. First, we attempted to capture all relevant articles by following the recommendations of the Preferred Reporting Items for Systematic Reviews and Meta-Analyses (PRISMA) statement. Nevertheless, this approach may have missed some articles. Second, it was not possible to quantitatively pool the outcomes from different studies because of heterogeneity inherent to the studies (e.g., study population, intervention components, and outcome measures). As such, we decided to present the findings of the study qualitatively. Furthermore, because our review relied on published articles alone, there may be unpublished findings that were not included in this review. This may raise the risk of publication bias. In addition, three studies included in this review [14,16,32] employed a time frame of less than 6 months, which could arguably be considered as pilot studies. Accordingly, these three studies could be excluded in the review in accordance with our inclusion/exclusion criteria. However, exclusion of these three studies did not affect our findings that pharmacist-led digital interventions were cost-effective. That is, digital interventions based on telephone, computer, and multiple technologies were still considered to be cost-effective even when these three studies were excluded in our review. Finally, this review included 14 studies only. Given the increasing attention in digital health, many studies are expected to be performed in the future to investigate the economic value of a diverse range of pharmacist-led digital interventions. As such, future literature reviews including a larger number of studies are necessary to confirm cost-effectiveness of such interventions. 

## 5. Conclusions

This review found evidence supporting the short-term cost-effectiveness of pharmacist-led digital interventions. Compared with traditional usual care, all interventions using telephones, computers, web-based interventions, or smartphones yielded more economic benefits. A video-based intervention was found cost-ineffective possibly because the intervention was not intensive enough to improve treatment adherence. This finding highlights the importance of aligning the design of an intervention with its purpose. 

Economic evaluation of digital interventions can contribute to evidence-based clinical decision making. Clinicians can use evidence from such economic evaluations to decide whether to adopt technology tools in their practice. Additionally, such evidence can be considered in a value-based reimbursement system since economic value is one of the important determinants that influence access to care. 

## Figures and Tables

**Table 1:** The Quality of Health Economics Studies (QHES) instrument.

Questions	Points
1. Was the study objective presented in a clear, specific, and measurable manner?	7
2. Was the perspective of the analysis (societal, third-party payer, etc.) stated and were reasons for its selection either stated *or implied*? ^a,^^b^	4
3. Were variable estimates used in the analysis from the best available source (i.e., randomized control trial—best, expert opinion—worst)? ^c^	8
4. If estimates came from a subgroup analysis, were the groups prespecified at the beginning of the study?	1
5. Was uncertainty handled by statistical analysis to address random events or sensitivity analysis to cover a range of assumption?	9
6. Was incremental analysis performed between alternatives for resources and costs?	6
7. Was the methodology for data abstraction (including the value of health states *for cost-utility analysis* and other benefits) stated? ^b^	5
8. Did the analytic horizon allow time for all relevant and important outcomes? Were benefits and costs that went beyond 1 year discounted (3% to 5%) and justification given for the discount rate *if the discount rate was arbitrarily determined*? ^b^	7
9. Was the measurement of costs appropriate and the methodology for the estimation of quantities, unit costs, and *price years* clearly described? ^a,^^b^	8
10. Were the primary outcome measure(s) for the economic evaluation clearly stated and did they include the major short-term, long-term, and negative outcomes? ^a^	6
11. Were the health outcomes measures/scales valid and reliable? If previously tested valid and reliable measures were not available, was justification given for the measures/scales used?	7
12. Were the *traditional* economic model (including structure), study methods and analysis, and the components of the numerator and denominator displayed in a clear, transparent manner? ^a,^^b^	8
13. Were the choice of *the traditional* economic model, main assumptions *of the traditional economic model*, and limitations of the study stated and justified? ^a,^^b^	7
14. Did the author(s) explicitly discuss direction and magnitude of potential biases? ^a^	6
15. Were the conclusions/recommendations of the study justified and based on the study results?	8
16. Was there a statement disclosing the source of funding for the study?	3
Total points	100

^a^ Weighted partial points were awarded if a study yielded at least one “yes” to the questions in the item. ^b^ Authors’ modifications are indicated by italics. ^c^ Weighted partial points were awarded if variable estimates were obtained from several sources with different qualities.

**Table 2:** Characteristics of the included economic evaluations.

Author (Year)	Type of Analysis	Country	Perspective	Currency	Economic Model	Time Horizon	Discount Rate	Funding Source
Amador-Fernandez et al. (2021) [15]	CUA	Spain	Patient and health system	EUR	No model (data directly from a clinical trial)	6 months	Not needed	Yes (Public)
Avery et al. (2012) [13]	CEA	United Kingdom	UK National Health Service	GBP	Simple probabilistic decision-analytic model	6 months	Not needed	Yes (Public)
Bosmans et al. (2007) [34]	CEA	Netherland	Societal	EUR	No model (data directly from a clinical trial)	6 months	Not needed	Yes (Public)
Dehmar et al. (2018) [35]	CEA	United States	Health service	USD	No model (data directly from a clinical trial)	12 months	Not needed	Yes (Public)
Dineen-Griffin et al. (2020) [16]	CUA	Australia	Societal	AUD	Decision tree	14 days	Not needed	Yes (Public)
Faleh AlMutairi et al. (2021) [32]	CEA	Saudi Arabia	Health service provider	SAR	No model (data from retrospective chart review)	4 months	Not needed	No
Fishman et al. (2013) [12]	CEA	United States	Health plan	USD	No model (data directly from a clinical trial)	Lifetime	3%, 5%, 7%	Yes (Public)
Hope et al. (2003) [14]	CEA	United States	Not reported	USD	Not reported	4 months	Not needed	Yes (Public)
Lazaro Cebas et al. (2022) [36]	CBA	Spain	Not reported	EUR	No model (data directly from a clinical trial)	9 months	Not needed	Yes (Public)
Lowres et al. (2014) [37]	CUA	Australia	Australian health funder	AUD	Not reported	10 years	5%	Yes (Private and Public)
Margusino-Framinan et al. (2022) [33]	CMA	Spain	Patient and societal	EUR	No model (data directly from a clinical trial)	12 months	Not needed	No
Padwal et al. (2018) [38]	CUA	Canada	Health care payer	CAD	Markov model	Lifetime	1.5%	Yes (Public)
Painter et al. (2017) [19]	CUA	United States	Payer	USD	No model (data directly from a clinical trial)	12 months	Not needed	Yes (Public)
Pyne et al. (2010) [20]	CUA	United States	Veterans health administration	USD	No model (data directly from a clinical trial)	6 and 12 months	Not needed	Yes (Public)

CEA: cost-effectiveness analysis; CUA: cost–utility analysis; CBA: cost–benefit analysis; CMA: cost-minimization analysis.

**Table 3:** Summary of the included cost-effectiveness analysis studies.

Author	Study Population	Intervention	Comparator	Types of Costs Included	Cost: Intervention vs. Comparator	Effectiveness: Intervention vs. Comparator	Incremental Cost-Effectiveness Ratio (ICER) ^a^
Telephone-based intervention							
Dehmer et al. [35]	Patients with hypertension	Telemonitoring of blood pressure	Usual care	Clinic-based (office visit, laboratory, radiology), pharmacy, and hospital costs	Change from baseline: USD −186 vs. USD 96	Incremental % of achieving blood pressure control for the intervention: 18.4% ^b^	USD 7337 per person achieving blood pressure control
Faleh AlMutairi et al. [32]	Patients with diabetes	Telemedicine care	Traditional care	Medications, laboratory tests, medical supplies, shipping, phone calls, and clinical visits	Saudi Riyal (SAR) 4820 vs. SAR 4151	Difference in HbA1c: 1.82 vs. 1.54	SAR 2373 per 1% reduction in the level of HbA1c
Lazaro Cebas et al. [36]	Polymedicated elderly patients aged ≥65 years	Phone call follow-up after discharge	No follow-up	Clinical pharmacist salary, cost per admission in elderly patients	Incremental cost for the intervention to prevent one readmission: EUR 3091 ^b^	30-day hospital readmission: 16.43% vs. 20.13%	Total cost saving: EUR 1301
Margusino-Framinan et al. [33]	Patients with HIV	Pre-post design (Intervention: teleconsultation with home drug delivery/mail-order pharmacy)		Direct (transportation, hospital pharmacy consultation service, home drug delivery) cost and indirect (productivity) cost	Cost saving of EUR 137 per patient per year for the intervention	No significant difference in HIV viral load and CD4+ level after the intervention	EUR 137 patient/year costs-saved and 18.5 h/patient/year working time gained
Padwal et al. [38]	Patients with cerebrovascular disease	Home blood pressure telemonitoring with pharmacist case management	Usual care	Pharmacist and physician cost, blood pressure device cost, drug cost, etc.	CAD 21,640 vs. CAD 23,020	Quality-adjusted life year (QALY): 8.83 vs. 8.00	The intervention was dominant, achieving improved health at a reduced cost
Painter et al. [19]	Veterans with posttraumatic stress disorder (PTSD)	Telemedicine-based collaborative care	Usual care	Outpatient and pharmacy costs	Incremental cost for the intervention: USD 2495	Incremental QALY: 0.008	USD 185,565/QALY
Pyne et al. [20]	Patients with depression	Telemedicine-based collaborative care	Usual care	(Base-case analysis) Outpatient and drug costs (Secondary analysis) Base-case costs and inpatient costs	Incremental cost for the intervention was significant (β = USD 1528, *p* < 0.001)	Incremental QALY for the intervention was significant (β = 0.018, *p* = 0.04)	(Base-case analysis) USD 85,634/QALY (Secondary analysis) USD 111,999–USD 132,175/QALY
**Computer- or web-based intervention**							
Avery et al. [13]	Patients electronically prescribed from a general practitioner	Computer-generated feedback for medication errors, educational outreach, and dedicated support	Computer-generated feedback for medication errors	Costs for generating error reports, training pharmacists, meetings, and time spent in each practice outside meetings following up errors	GBP 1050 vs. GBP 93	Primary outcomes: (1) NSAID-related error: 3% vs. 4% (2) Beta blocker-related error: 2% vs. 3% (3) ACE inhibitor- or loop diuretic-related error: 5% vs. 8%	GBP 66 per error avoided
Fishman et al. [12]	Adults with hypertension alone (no diagnosis of diabetes, cardiovascular, or other serious conditions)	Home blood pressure monitoring + pharmacist’s web-based management of patients’ blood pressures	Home blood pressure monitoring	Physical and human resources used to provide an intervention or usual care	USD 400 vs. USD 67	Discounted change in life expectancy: (1) Women: 0.44 vs. 0.29 (2) Men: 0.53 vs. 0.35	(1) Women: USD 2220/year (2) Men: USD 1850/year
Hope et al. [14]	Adults with outpatient appointment at ambulatory care clinics	Tiered review method (computer-based review before a clinician’s review)	Traditional pharmacist review method	Training cost, data analyst, nurse, and pharmacist	USD 22,606 vs. USD 44,580	777 adverse drug events (ADEs) and 666 medication errors (MEs) ^b^	Cost per ADE identified: USD 42.40 with the tiered method vs. USD 68.70 with the traditional method
**Videotape-based intervention**							
Bosmans et al. [34]	Patients with a new prescription for a non-tricyclic antidepressant	Coaching program (three contacts with pharmacist and a take-home video)	Usual care	Direct medical costs and indirect costs	EUR 3275 vs. EUR 2961	(1) Adherence: No significant difference between the two groups (mean difference: 2.1%, 95% CI: −5.6 to 9.8) (2) Improvements in depressive symptoms measured by the Hopkins Symptom Checklist (SCL): No significant difference between the two groups (mean difference: −0.15, 95% CI: −0.54 to 0.23)	(1) EUR 149 per 1% improvement in adherence (2) EUR 2550 per point improvement on the SCL
**Smartphone-based intervention**							
Lowres et al. [37]	Patients aged 65 or older with no severe medical condition	Screening of atrial fibrillation (AF) using iPhone electrocardiogram (iECG)	No screening of AF	Diagnostic assessment of AF costs, anticoagulation, and monitoring costs	Not reported separately for the intervention and comparator	Not reported separately for the intervention and comparator	AUD 5988/QALY and AUD 30,481 for preventing one stroke
**Multiple technologies system-based intervention**							
Amador-Fernandez et al. [15]	Patients presenting minor ailments or requesting a non-prescription medication for minor ailments	Face-to-face consultation on a web-based program plus telephone follow-up	Usual care	Health professionals’ consultation time, medication costs, pharmacists’ training costs, and investment of the pharmacy and consultation costs	EUR 20 vs. EUR 13	QALY: 0.0248 vs. 0.0245	EUR 24,733/QALY
Dineen-Griffin et al. [16]	Patients with minor ailments	Face-to-face consultation using the technology-integrated platforms plus telephone follow-up	Usual care	Direct costs	(Base-case analysis) AUD 27 vs. AUD 20 (Multi-way sensitivity analysis)AUD 34 vs. AUD 23	(Base-case analysis) QALY: 0.0296 vs. 0.0264 (Multi-way sensitivity analysis) QALY: 0.0296 vs. 0.0264	(Base-case analysis) AUD 2277/QALY (Multi-way sensitivity analysis) AUD 3502/QALY

^a^ Each study determined cost-effectiveness of digital interventions by comparing ICERs to a willingness-to-pay threshold. ^b^ Not separately reported for the intervention and comparator groups.

**Table 4:** Results of the study quality.

QHES Item	Amador-Fernandez et al. [15]	Avery et al. [13]	Bosmans et al. [34]	Dehmer et al. [35]	Dineen-Griffin et al. [16]	Faleh AlMutairi et al. [32]	Fishman et al. [12]	Hope et al. [14]	Lazaro Cebas et al. [36]	Lowres et al. [37]	Margusino-Framinan et al. [33]	Padwal et al. [38]	Painter et al. [19]	Pyne et al. [20]
1	FP	FP	FP	FP	FP	FP	FP	FP	FP	FP	FP	FP	FP	FP
2	PP	PP	PP	PP	FP	PP	PP	NP	NP	PP	FP	PP	PP	PP
3	FP	FP	FP	FP	FP	FP	FP	FP	FP	FP	FP	FP	FP	FP
4	FP	FP	NA	NA	NA	NA	NA	NA	FP	NA	NA	NA	FP	FP
5	FP	FP	FP	FP	FP	FP	FP	NP	FP	FP	FP	FP	FP	FP
6	FP	FP	FP	FP	FP	FP	FP	FP	FP	FP	NP	FP	FP	FP
7	FP	FP	FP	FP	FP	FP	FP	FP	FP	FP	FP	FP	FP	FP
8	NA	NA	NA	NA	NA	NA	FP	NA	NA	FP	NA	FP	NA	NA
9	FP	PP	FP	PP	FP	PP	FP	PP	PP	FP	NP	FP	PP	FP
10	PP	PP	PP	PP	PP	PP	PP	PP	PP	FP	PP	FP	PP	PP
11	FP	FP	FP	FP	FP	FP	FP	FP	FP	FP	FP	FP	FP	FP
12	PP	PP	PP	PP	FP	PP	PP	PP	PP	PP	PP	FP	PP	PP
13	PP	PP	PP	PP	FP	PP	PP	PP	PP	PP	PP	FP	PP	PP
14	PP	PP	FP	NP	NP	NP	PP	NP	NP	PP	PP	NP	NP	NP
15	FP	FP	FP	FP	FP	FP	FP	FP	FP	FP	FP	FP	FP	FP
16	FP	FP	FP	FP	FP	FP	FP	FP	FP	FP	NP	FP	FP	FP
Quality	Good	Good	Good	Fair	Good	Fair	Good	Fair	Fair	Good	Fair	Good	Fair	Good

QHES: Quality of Health Economic Studies; FP: full points; PP: partial points; NP: no points; NA: not applicable.

## Data Availability

Not applicable.
